# HLA-dependent association between EBNA1 antibody levels and disability progression in multiple sclerosis

**DOI:** 10.1136/jnnp-2026-338964

**Published:** 2026-07-31

**Authors:** Anna Karin Hedström, Olivia Thomas, Pernilla Strid, Jan Hillert, Ingrid Kockum, Tim Waterboer, Tomas Olsson, Lars Alfredsson

**Affiliations:** 1 Department of Clinical Neuroscience, Karolinska Institutet, Stockholm, Sweden; 2 Department of Clinical Neuroscience, Karolinska Institute, Stockholm, Sweden; 3 Therapeutic Immune Design, Department of Clinical Neuroscience, Karolinska Institute, Stockholm, Sweden; 4 German Cancer Research Centre, Heidelberg, Baden-Württemberg, Germany; 5 Karolinska Institutet, Stockholm, Stockholm County, Sweden

**Keywords:** MULTIPLE SCLEROSIS, DEMYELINATING DISEASES, NEUROIMMUNOLOGY, Prognosis

## Abstract

**Background:**

Epstein-Barr virus (EBV) is strongly implicated in the development of multiple sclerosis (MS) but whether EBV-related immune responses are also relevant for disease progression after diagnosis remains unclear. We investigated whether Epstein-Barr nuclear antigen 1 (EBNA1) antibody levels are associated with disability progression in MS and whether this association is modified by human leucocyte antigen (HLA)-A*02:01 and HLA-DRB1*15:01.

**Methods:**

We analysed 5706 patients with MS from two population-based Swedish studies with longitudinal follow-up in the Swedish MS registry. EBNA1 IgG levels were analysed as a continuous variable, expressed per one SD increase. The primary outcome was confirmed disability worsening (CDW). Cox proportional hazards models were used to estimate hazard ratios (HRs) with 95% confidence intervals (CIs). Effect modification by HLA-A*02:01 and DRB1*15:01 was assessed using interaction terms and stratified analyses. Secondary and sensitivity analyses included dichotomisation of EBNA1 levels, alternative progression outcomes, delayed-entry models and treatment-related analyses.

**Results:**

Higher EBNA1 antibody levels were associated with a reduced risk of CDW among individuals carrying both A*02:01 and DRB1*15:01 (HR 0.87, 95% CI 0.81 to 0.93), whereas associations were weaker or absent in other HLA strata. Findings were similar in secondary and sensitivity analyses.

**Conclusion:**

Our findings suggest that EBV-related antibody responses may have prognostic relevance for MS progression in specific genetic contexts, highlighting the importance of host-virus interactions beyond disease onset.

**WHAT IS ALREADY KNOWN ON THIS TOPIC**

**WHAT THIS STUDY ADDS**

- In this population-based cohort study, higher EBNA1 antibody levels were associated with a lower risk of disability progression, with the strongest inverse association observed among individuals carrying both HLA-DRB1*15:01 and HLA-A*02:01. The findings suggest that the association between EBV-related antibody responses and MS progression may vary according to host immunogenetic background.

**HOW THIS STUDY MIGHT AFFECT RESEARCH, PRACTICE OR POLICY**

- The results support more genetically stratified approaches in studies of biomarkers and disease progression in MS and suggest that EBV-related immune markers should not be interpreted uniformly across patient groups. They also motivate further mechanistic and longitudinal studies of host–virus interactions in relation to long-term disease course.

## Background

 Multiple sclerosis (MS) is a chronic inflammatory disease of the central nervous system characterised by substantial heterogeneity in clinical course. While several genetic and environmental factors contributing to MS susceptibility have been identified,^1 2^ considerably less is known about determinants of disease progression after onset. Identifying prognostic biomarkers that reflect disease-relevant biological processes remains a major unmet need.

Epstein-Barr virus (EBV) infection is now widely considered a prerequisite for MS development, supported by strong epidemiological and mechanistic evidence.^3 4^ Elevated antibody responses to EBV antigens, including Epstein-Barr virus nuclear antigen (EBNAs), are consistently associated with increased MS risk.^3 4^ These findings have been interpreted within several non-mutually exclusive frameworks, including molecular mimicry and altered immune regulation of EBV-infected B cells.^5–7^ However, whether EBV-related immune responses are also relevant for MS progression after onset is unclear, and available data are limited and inconsistent.^8–12^

Virus-specific antibody responses are partly heritable and strongly influenced by HLA variation,^5^ indicating that EBV serological markers reflect host immunogenetic background as well as viral exposure. The HLA-DRB1*15:01 allele represents the strongest genetic risk factor for MS and shapes CD4+T cell antigen presentation, including responses to EBV-derived peptides.^3^ Conversely, HLA-A*02:01 is consistently associated with reduced risk of MS and reduced risk of infectious mononucleosis.^5^ Although its protective effect has often been attributed to efficient presentation of immunodominant EBV epitopes to CD8+T cells^13 14^, the HLA region is characterised by extensive linkage disequilibrium, and broader immunoregulatory mechanisms, including variation in interferon signalling pathways, may also contribute.^15^ Together, these observations suggest that the interpretation of EBV-related immune markers may depend on host immunogenetic background.

In this population-based cohort study, we investigated whether EBNA1 antibody levels measured during the disease course are associated with subsequent disability progression and whether this association is modified by HLA-A*02:01 and HLA-DRB1*15:01 status.

## Methods

The present study comprises patients recruited through two population-based studies in Sweden: Epidemiologic Investigation of Multiple Sclerosis (EIMS)^16^ and Genes and Environment in Multiple Sclerosis (GEMS).^17^ EIMS enrolled incident cases of MS from hospital-based neurology units between April 2005 and June 2015 (n=2880), whereas GEMS identified prevalent MS cases, distinct from those included in EIMS, through the Swedish MS registry between November 2009 and November 2011 (n=6148). Eligibility criteria for cases were age 16–70 years, residence in Sweden, and a neurologist-confirmed diagnosis of MS according to the McDonald criteria.^18 19^ The response rate was 93% among cases in EIMS and 82% among cases in GEMS. All participants filled out a standardised questionnaire on environmental exposures and lifestyle habits and were invited to provide blood samples. Serum samples were available for 2021 individuals from EIMS and for 4319 individuals from GEMS. Of the 6340 patients with available blood samples, 5706 (90%) had longitudinal Expanded Disability Status Scale (EDSS) data recorded in the Swedish MS registry and were included in the present study (eFigure 1).

Both studies were approved by the Regional Ethical Review Board at Karolinska Institute (reference numbers 2004–252/1-4 and 2008/1617-31/2) and conducted in accordance with the principles of Helsinki and its subsequent amendments.

### Genotyping and measurement of EBNA-1 IgG levels

HLA-DRB1 and HLA-A alleles were determined at four-digit resolution. Genotyping was performed on the MS replication chip, an Illumina exome-based array supplemented with approximately 90 000 custom markers, including high-density coverage of the HLA region. Classical HLA alleles were subsequently imputed using HLA*IMP:02.^20^ IgG antibodies against EBNA1 were measured using multiplex serology targeting the EBNA-1 peptide segment encompassing amino acids 385–420^5^. This peptide has previously been identified as the EBNA-1 region most strongly associated with MS susceptibility.^18^ The multiplex serology platform has previously demonstrated high analytical reproducibility (R² = 0.97) and low imprecision (median coefficient of variation≤5.4%).^5^ All samples were analysed using the same assay platform and standardised laboratory procedures. Antibody levels were quantified using dual-laser, flow-based detection and expressed as median fluorescence intensity (MFI) units. All included samples showed detectable reactivity against the EBNA1 385–420 peptide, although antibody levels varied substantially across individuals.

### Outcome measures

The Swedish MS registry is used in nearly all neurology units nationwide and longitudinal data are continuously recorded for each patient. The registry includes information on disease-modifying treatment (DMT), clinical disease activity and physical disability.

To assess disability progression over time, baseline was defined as the date of the first recorded EDSS assessment at or after the time of MS diagnosis. Confirmed disability worsening (CDW) was defined as an increase in EDSS sustained for at least 6 months between consecutive assessments. The required magnitude of increase depended on baseline EDSS: ≥1.5 points for baseline EDSS=0, ≥1.0 point for baseline EDSS<5.5 and ≥0.5 points for baseline EDSS≥5.5. Improvements in EDSS did not contribute to the outcome definition. Time to disability milestones EDSS 3 and EDSS 4 were examined as secondary outcomes and were limited to subgroups of patients with a baseline EDSS below 3.

To facilitate interpretation of the analytical framework and the use of longitudinal EDSS data across the different analyses, a schematic overview is provided in eFigure 2.

### Statistical analysis

Baseline characteristics were summarised using descriptive statistics, with continuous variables presented as means with SD or medians with IQRs, as appropriate, and categorical variables as counts and percentages.

Time-to-event analyses were conducted using Cox proportional hazards regression, with time since baseline as the underlying time scale. EBNA antibody levels were analysed as continuous variables and modelled per one SD increase to facilitate interpretation and comparability of effect estimates. HRs with 95% CIs were reported. The proportional hazards assumption was assessed using Schoenfeld residuals and no violations were detected. Individuals were followed until the occurrence of the outcome event, drop-out, death or end of follow-up (22 April 2022), whichever occurred first. The primary analysis assessed the association between EBNA1 antibodies and risk of CDW in the total cohort, adjusted for sex, age at baseline, calendar year of diagnosis, disease phenotype, disease duration and baseline EDSS. Effect modification by HLA-A*02:01 and HLA-DRB1*15:01 was evaluated by including multiplicative interaction terms between EBNA1 levels and HLA status in the Cox model. In addition, stratified analyses were performed across four predefined HLA subgroups defined by combined HLA-A*02:01 and HLA-DRB1*15:01 status. For visualisation, restricted cubic spline curves were generated to illustrate the association between EBNA1 levels and risk of CDW. Two alternative reference scales were used. First, subgroup-specific median EBNA1 levels were used as reference within each HLA stratum, allowing assessment of relative risk gradients within genetic subgroups. Second, the median EBNA1 level in the total cohort was used as a common reference to facilitate comparison of absolute risk levels across HLA strata.

Secondary analyses examined EBNA1 levels dichotomised at the cohort median. Using this categorisation, associations with CDW as well as with time to EDSS milestones 3 and 4 were evaluated in the total cohort and within each HLA subgroup.

Given the potential influence of DMTs on EBNA1 antibody levels, several treatment-related sensitivity analyses were performed. These included analyses restricted to individuals treated exclusively with platform therapies (interferon-beta, glatiramer acetate or teriflunomide), analyses restricted to treated individuals with adjustment for DMT exposure as a time-varying covariate and analyses additionally adjusted for DMT status at the time of EBNA1 sampling (none, platform or high-efficacy therapy). All sensitivity analyses were otherwise specified identically to the primary analysis.

To address potential bias related to variation in timing of EBNA measurement during the disease course, a sensitivity analysis was conducted using delayed entry (left truncation), with follow-up starting at the date of EBNA sampling. This analysis included incident cases and otherwise mirrored the primary model specification. In an additional sensitivity analysis, the delayed-entry model was further adjusted for educational attainment (pre-secondary, secondary or post-secondary education), other autoimmune disease (yes vs no), infectious mononucleosis (yes, no or unsure), smoking status (current smoker vs non-smoker), alcohol consumption (no consumption, low, moderate or high consumption according to cutoffs used by Statistics Sweden), body mass index (<25, 25–30 or >30 kg/m^2^), sun exposure and physical activity. Sun exposure habits were assessed using two four-level items, combined into an index ranging from 2 to 8 points and dichotomised at the median (low <5 vs high >6). Physical activity was assessed through four predefined categories, from predominantly sedentary behaviour to regular sweat-inducing exercise more than three times per week. In a subsample (n=3829), we used ancestry-informative principal components to adjust for population stratification. In this analysis, population outliers were excluded (n=85). To provide additional context regarding the timing of blood sampling, we examined the correlation between time from baseline to blood sampling and EBNA1 antibody levels. Finally, we identified the most recent EDSS score prior to blood sampling and further adjusted the models for disability status at the time of biomarker assessment. This analysis was performed to evaluate whether the observed associations between EBNA1 antibody levels and subsequent disability progression could be explained by differences in disability status at the time of biomarker assessment. All analyses were conducted in SAS version 9.4.

## Results

The study included 5706 patients with MS who were followed longitudinally in the Swedish MS registry. Baseline characteristics stratified by HLA-DRB1*15:01 and HLA-A*02:01 status are shown in table 1. Age at disease onset and diagnosis differed modestly across HLA strata, with the highest age at onset observed among DRB1*15:01*-*negative*,* A*02:01-positive individuals (p<0.001). Nordic ancestry was more common among DRB1*15:01*-*positive patients and among A*02:01 carriers (p<0.0001).

**Table 1:** Characteristics of overall sample and by HLA-DRB1*15:01 and HLA-A*02:01 status

		Total	DRB1*15:01- A*02:01-	DRB1*15:01- A*02:01+	DRB1*15:01+A*02:01-	DRB1*15:01+A*02:01+	P value
N		5706	1472	951	1815	1468	
Age at disease onset (SD)		33.9 (10.6)	33.9 (10.6)	35.5 (10.5)	33.4 (10.5)	33.7 (10.7)	0.0001
Age at diagnosis (SD)		38.6 (11.2)	38.7 (11.2)	39.8 (11.3)	38.1 (11.1)	38.3 (11.3)	0.002
Sex, n (%)	Female	4163 (73.0)	1046 (71.1)	670 (70.4)	1366 (75.3)	1081 (73.6)	0.01
	Male	1543 (27.0)	426 (28.9)	281 (29.6)	449 (24.7)	387 (26.4)	
Ancestry, n (%)	Nordic	5036 (88.3)	1205 (81.9)	827 (87.0)	1634 (90.0)	1370 (93.3)	<0.0001
	Non-Nordic	670 (14.6)	267 (18.1)	124 (13.0)	181 (10.0)	98 (6.7)	
MS type, n (%)	Relapsing-onset	5103 (89.4)	1321 (89.7)	826 (86.9)	1639 (90.3)	1317 (89.7)	0.05
	Progressive-onset	135 (9.2)	126 (8.6)	106 (11.2)	158 (8.7)	135 (9.2)	
	Unknown	16 (1.4)	25 (1.7)	19 (2.0)	18 (1.0)	16 (20.5)	
DMT, n (%)	Never	1548 (27.1)	370 (25.1)	265 (27.9)	489 (26.9)	424 (28.9)	0.19
	Platform	2523 (44.2)	653 (44.4)	430 (45.2)	811 (44.7)	629 (42.9)	
	High-efficacy	340 (6.0)	91 (6.2)	51 (5.4)	97 (5.3)	101 (6.9)	
	Escalation*	1295 (22.7)	358 (24.3)	205 (21.6)	418 (23.0)	314 (21.4)	
Time to DMT initiation, years (SD)		8.3 (9.1)	8.1 (8.9)	7.6 (8.6)	8.6 (9.2)	8.8 (9.5)	0.02
Baseline EDSS, mean (SD) Baseline EDSS, median (IQR)		2.9 (2.1) 2.5 (1.0–4.0)	2.8 (2.1) 2.5 (1.0–4.0)	2.8 (2.1) 2.5 (1.0–4.0)	2.8 (2.1) 2.0 (1.0–4.0)	2.9 (2.1) 2.5 (1.0–4.0)	0.002
EBNA1 IgG levels (MFI)†		6883 (3092)	6514 (3179)	6258 (3346)	7447 (2907)	6960 (2924)	<0.0001
Past infectious mononucleosis, n (%)	Yes	725 (12.7)	213 (14.5)	93 (9.8)	262 (14.4)	157 (10.7)	0.0006
	No	4093 (71.7)	1024 (69.6)	710 (74.7)	1269 (69.9)	1090 (74.3)	
	Unsure	888 (15.6)	235 (16.0)	148 (15.6)	284 (15.7)	221 (15.1)	
Smoking, n (%)	Current smoker	1914 (33.5)	487 (33.1)	326 (34.3)	602 (33.2)	499 (34.0)	0.89
	Non-smoker	3792 (66.5)	985 (66.9)	625 (65.7)	1213 (66.8)	969 (66.0)	

* initial platform therapy followed by high-efficacy DMT.

† measured as median fluorescence intensity.

DMT, disease-modifying therapy; EBNA, Epstein-Barr nuclear antigen ; EDSS, Expanded Disability Status Scale; HLA, human leucocyte antigen; MS, multiple sclerosis.

EBNA-1 IgG levels differed markedly by HLA status, with lower levels observed among A02:01-positive patients and higher levels among DRB1*15:01-positive patients (p<0.0001). Baseline EDSS was similar across groups, although small differences were observed (p=0.002). The distribution of disease-modifying therapy exposure did not differ substantially between HLA strata. Higher EBNA1 levels were associated with a reduced risk of CDW in the overall cohort (HR per 1 SD increase 0.93 (0.90–0.96) (table 2, figure 1). Evidence for effect modification by DRB1*15:01 was observed (p for interaction=0.04), with additional evidence for three-way interaction with A*02:01 (p=0.03).

**Figure 1:**
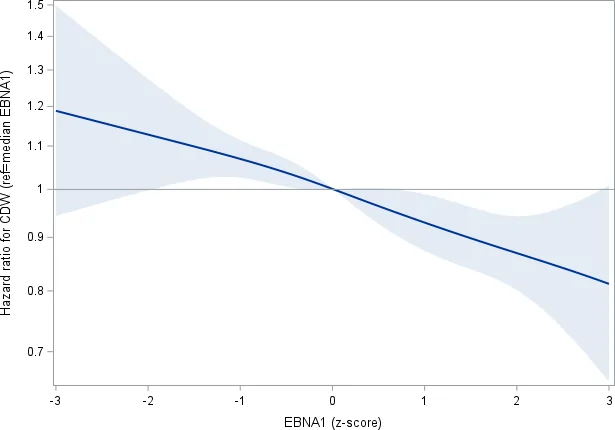
Association between EBNA1 levels and risk of confirmed disability progression. Hazards ratios for confirmed disability progression across standardised EBNA1 levels are shown relative to the median EBNA1 value (HR=1). EBNA1 levels were standardised to z-scores. The solid line represents adjusted HRs estimated from Cox proportional hazards models; the shaded area indicates 95% CIs. Models were adjusted for sex, age at baseline, ancestry, calendar year of diagnosis, disease phenotype, disease duration, baseline EDSS, HLA-DRB1*15:01 status and HLA-A*02:01 status. EBNA, Epstein Barr virus nuclear antigen; EDSS, expanded disability status scale; CDW, confirmed disability worsening; HLA, human leucocyte antigen.

**Table 2:** Association between EBNA1 levels and risk of confirmed disability progression, overall and stratified by HLA-A*02:01 and HLA-DRB1*15:01 status.

DRB1*15:01	A*02:01	N	Years (SD)	Outcome (%)	HR (95% CI)*	HR (95% CI)†
-/+	-/+	5706	6.7 (5.1)	3510 (61.5)	**0.93** **(0.90 to 0.96**)	**0.93** **(0.90 to 0.96**)
–	–	1472	6.6 (5.1)	897 (60.7)	**0.92** **(0.86 to 0.98**)	**0.93** **(0.97 to 0.99**)
–	+	951	6.9 (5.0)	589 (61.9)	0.96 (0.89 to 1.03)	0.97 (0.90 to 1.04)
+	–	1815	6.7 (5.2)	1121 (61.8)	0.97 (0.91 to 1.03)	0.97 (0.91 to 1.04)
+	+	1468	6.8 (5.2)	903 (61.5)	**0.86** **(0.80 to 0.92**)	**0.87** **(0.81 to 0.93**)

Bold values indicate statistical significance.

EBNA2 levels were standardized to z-scores; HRs represent the relative hazard per 1 standard deviation increase in EBNA2.

* unadjusted.

† adjusted for sex, age at baseline, ancestry, calendar year of diagnosis, disease phenotype, disease duration, and baseline EDSS

DMT, disease-modifying treatment; EBNA, Epstein Barr virus nuclear antigen; EDSS, expanded disability status scale; HLA, human leucocyte antigen.;

When stratified by HLA status, the association differed across genetic subgroups. The strongest inverse association was observed among participants carrying both A*02:01 and DRB1*15:01 (HR 0.87, 95% CI 0.81 to 0.93), whereas no clear or only weak associations were observed in the other HLA strata (table 2). Figure 2 displays subgroup-specific associations using HLA-stratum-specific median EBNA1 levels as reference, whereas eFigure 3 presents the same associations using the median EBNA1 level of the total cohort as a common reference.

**Figure 2:**
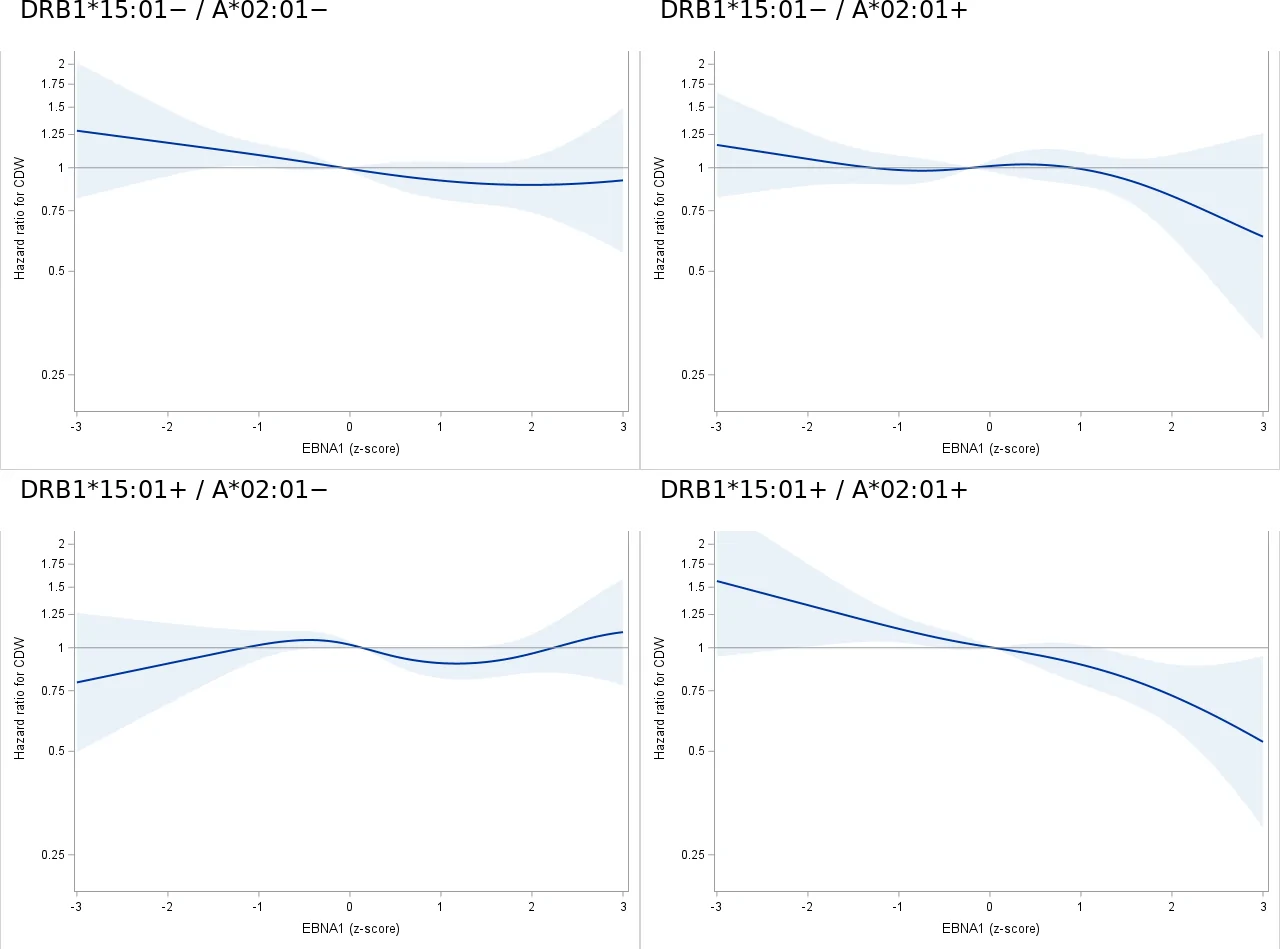
Association between EBNA1 levels and risk of confirmed disability progression, stratified by HLA status. HRs for confirmed disability progression across standardised EBNA1 levels are shown relative to the median EBNA1 value (HR=1). EBNA1 levels were standardised to z-scores. The solid line represents adjusted HRs estimated from Cox proportional hazards models; shaded area indicates 95% CIs. Models were adjusted for sex, age at baseline, ancestry, calendar year of diagnosis, disease phenotype, disease duration and baseline EDSS. CDW, confirmed disability worsening; EBNA, Epstein Barr virus nuclear antigen; EDSS, expanded disability status scale.

When EBNA1 levels were dichotomised at the median, higher EBNA1 levels were associated with a reduced risk of CDW in the overall cohort (HR 0.91, 95% CI 0.85 to 0.97) (table 3). Stratified analyses revealed marked heterogeneity by HLA genotype. The strongest and most consistent inverse associations were observed among patients carrying both DRB1*15:01 and A*02:01, for whom higher EBNA1 levels were associated with reduced risks of CDW (HR 0.88, 95% CI 0.77 to 1.00), EDSS 3 (HR 0.76, 95% CI 0.61 to 0.94) and EDSS 4 (HR 0.79, 95% CI 0.64 to 0.98). In contrast, among participants lacking either DRB1*15:01 or A*02:01, point estimates were closer to unity and CIs included one for all outcomes (table 3).

**Table 3:** Association between EBNA1 status and risk of confirmed disability progression, overall and stratified by HLA-A*02:01 and HLA-DRB1*15:01 status.

Confirmed disease worsening							
DRB1*15:01	A*02:01	EBNA1	N	Years (SD)	Outcome (%)	HR (95% CI)*	HR (95% CI)†
-/+	-/+	Low	2853	6.6 (5.2)	1789 (62.7)	1.0 (reference)	1.0 (reference)
		High	2853	6.9 (5.1)	1721 (60.3)	**0.90 (0.84 to 0.96**)	**0.91 (0.85 to 0.97**)
–	–	Low	803	6.4 (5.2)	501 (62.4)	1.0 (reference)	1.0 (reference)
		High	669	6.7 (5.0)	396 (59.2)	0.88 (0.77 to 1.00)	0.89 (0.78 to 1.01)
–	+	Low	546	6.9 (5.1)	341 (62.5)	1.0 (reference)	1.0 (reference)
		High	405	6.9 (4.8)	248 (61.2)	0.98 (0.83 to 1.15)	0.99 (0.84 to 1.17)
+	–	Low	782	6.4 (5.1)	488 (62.4)	1.0 (reference)	1.0 (reference)
		High	1033	7.0 (5.3)	633 (61.3)	0.91 (0.81 to 1.02)	0.92 (0.81 to 1.03)
+	+	Low	722	6.6 (5.2)	459 (63.6)	1.0 (reference)	1.0 (reference)
		High	746	7.0 (5.2)	444 (59.2)	**0.87 (0.77 to 0.99**)	**0.88 (0.77 to 1.00**)
**EDSS 3**							
DRB1*15:01	A*02:01	**EBNA1**	**N**	**Years (SD)**	**Outcome (%)**	**HR (95% CI)** *	**HR (95% CI)** †
-/+	-/+	Low	1370	8.4 (5.5)	660 (48.2)	1.0 (reference)	1.0 (reference)
		High	1664	8.7 (5.5)	715 (43.0)	**0.86 (0.77 to 0.96**)	**0.87 (0.79 to 0.97**)
–	–	Low	397	8.4 (5.7)	181 (45.6)	1.0 (reference)	1.0 (reference)
		High	402	8.7 (5.4)	158 (39.3)	0.82 (0.67 to 1.02)	0.84 (0.68 to 1.04)
–	+	Low	265	8.7 (5.3)	120 (45.3)	1.0 (reference)	1.0 (reference)
		High	249	8.5 (5.2)	103 (41.4)	0.90 (0.70 to 1.18)	0.95 (0.73 to 1.24)
+	–	Low	379	8.4 (5.7)	191 (50.4)	1.0 (reference)	1.0 (reference)
		High	613	8.7 (5.6)	286 (46.7)	0.91 (0.75 to 1.09)	0.93 (0.78 to 1.12)
+	+	Low	329	8.3 (5.4)	168 (51.1)	1.0 (reference)	1.0 (reference)
		High	400	8.7 (5.5)	168 (42.0)	**0.78 (0.63 to 0.97**)	**0.76 (0.61 to 0.94**)
**EDSS 4**							
DRB1*15:01	A*02:01	**EBNA1**	**N**	**Years (SD)**	**Outcome (%)**	**HR (95% CI)** *	**HR (95% CI)** †
-/+	-/+	Low	1831	9.6 (5.8)	676 (36.9)	1.0 (reference)	1.0 (reference)
		High	2076	9.9 (5.5)	657 (31.7)	**0.83 (0.74 to 0.92**)	**0.89 (0.80 to 0.99**)
–	–	Low	524	9.5 (5.9)	190 (36.3)	1.0 (reference)	1.0 (reference)
		High	501	9.5 (5.3)	158 (31.5)	0.87 (0.70 to 1.08)	0.95 (0.77 to 1.18)
–	+	Low	359	9.6 (5.7)	124 (34.5)	1.0 (reference)	1.0 (reference)
		High	311	9.8 (5.5)	86 (27.7)	0.78 (0.60 to 1.02)	0.86 (0.65 to 1.14)
+	–	Low	498	9.6 (5.8)	184 (37.0)	1.0 (reference)	1.0 (reference)
		High	758	10.1 (5.7)	250 (33.0)	0.86 (0.71 to 1.04)	0.94 (0.78 to 1.14)
+	+	Low	450	9.5 (5.9)	178 (39.6)	1.0 (reference)	1.0 (reference)
		High	506	10.0 (5.5)	163 (32.2)	**0.78 (0.63 to 0.96**)	**0.79 (0.64 to 0.98**)

* unadjusted

† adjusted for sex, age at baseline, ancestry, calendar year of diagnosis, disease phenotype, disease duration, and baseline EDSS. Bold values indicate statistical significance.

DMT=disease-modifying treatment. EBNA, Epstein Barr virus nuclear antigen; EDSS, expanded disability status scale; HLA, human leucocyte antigen.;

The findings remained consistent across multiple sensitivity analyses. Similar inverse associations between EBNA1 levels and the risk of confirmed disability worsening were observed when analyses were restricted to patients treated exclusively with platform therapies (online supplemental table 1), when limited to DMT-treated patients with adjustment for treatment as a time-varying covariate (online supplemental table 2) and when additionally adjusting for treatment status at the time of blood sampling (online supplemental table 3). Furthermore, analyses restricted to incident cases with baseline defined as the date of blood sampling showed attenuation of the overall association, while the inverse association among individuals carrying both DRB1*15:01 and A*02:01 remained evident (online supplemental table 4). Additional adjustment for educational attainment, other autoimmune diseases, infectious mononucleosis, smoking, alcohol consumption, body mass index, sun exposure and physical activity did not materially alter the estimates (online supplemental table 5). The inverse association between EBNA1 levels and CDW remained consistent after exclusion of genetic outliers and additional adjustment for principal component vectors (HR per 1 SD increase 0.92 (0.88–0.96)). No meaningful correlation was observed between time from baseline to blood sampling and EBNA1 antibody levels (r=0.01, p=0.24). Disability status based on the most recent EDSS assessment prior to blood sampling was similar across HLA strata (online supplemental table 6). The median interval between this EDSS assessment and the blood sampling date was 0.9 years (IQR 0.3–2.5). Additional adjustment for EDSS prior to blood sampling attenuated the estimates slightly but did not materially alter the observed pattern (online supplemental table 7).

## Discussion

In this population-based cohort, higher EBNA1 antibody levels were associated with a lower risk of disability progression in MS among individuals carrying both DRB1*15:01 and A*02:01, while associations were weak or absent in other genetic strata. Interaction analyses indicated effect modification by HLA, suggesting that the prognostic relevance of EBNA1 antibody levels depends on host immunogenetic background.

While EBV infection is now widely regarded as a prerequisite for MS development,^21^ its relevance for disease progression remains uncertain. Prior studies examining EBV-related antibody levels in established MS have yielded inconsistent results, and few have evaluated potential modification by host genotype.^8–12^ Although the association between EBNA1 antibody levels and disability progression was modest and largely restricted to specific HLA genotypes, our findings do not exclude a potential contribution of EBV infection to the chronic progression of MS through mechanisms not captured by EBNA1 antibody responses. By integrating longitudinal registry data with immunological and genetic information, our study extends prior work by demonstrating that EBNA1 antibody levels carry prognostic information in a genetically restricted subgroup and that this pattern is consistent across multiple analytic approaches, including continuous and dichotomised EBNA1 measures and clinically relevant disability outcomes.

At first sight, the inverse association between EBNA1 antibody levels and disability progression may appear difficult to reconcile with the established role of EBV in MS susceptibility. One possible interpretation is that serum EBNA1 antibody levels reflect different biological processes in disease susceptibility and disease progression. Following disease onset, circulating EBNA1 antibody levels may primarily capture differences in host immune control of EBV rather than the degree of EBV-driven pathology. Consequently, lower EBNA1 levels would not necessarily indicate reduced EBV involvement, but rather an immune state characterised by less effective antiviral surveillance or altered B-cell function, which in turn could be associated with disability accumulation. Importantly, circulating EBNA1 antibody levels may not directly reflect immune processes occurring within the central nervous system. If EBV contributes to progression through compartmentalised CNS inflammation, serum EBNA1 levels are unlikely to represent a direct measure of such pathogenic activity. Instead, circulating EBNA1 antibody levels may function primarily as a biomarker of systemic immune status, which could explain why higher serum levels were associated with a more favourable disease course.

The stronger association observed among carriers of both DRB1*15:01 and A*02:01 suggests that the prognostic significance of EBNA1 antibody levels depends on immunogenetic context. Given the opposing effects of these alleles on EBNA1 responses, variation in antibody levels may be particularly informative in individuals in whom both genetic influences are present.

Since several DMTs can influence humoral immune responses, treatment-related influences represent an important consideration when interpreting EBNA1 antibody levels. Therapies targeting B cells or lymphocyte trafficking^22–24^ have been associated with reduced EBNA1 antibody levels, potentially through effects on antigen presentation, reduced B cell numbers, or broader immune regulation. Our sensitivity analyses addressing DMT in multiple ways yielded consistent patterns, reducing the likelihood that the observed associations are explained by treatment confounding. These findings support the interpretation that EBNA1 levels, at least in some genetic contexts, may capture host immune features relevant to long-term course rather than simply reflecting treatment exposure.

Several limitations should be acknowledged. A key concern when using antibody levels measured after MS onset is whether they reflect prior disease activity or severity rather than predicting future progression. We addressed this issue by conducting a delayed-entry sensitivity analysis, with follow-up initiated at the date of EBNA sampling, thereby reducing bias related to left truncation and aligning exposure assessment more closely with the risk period in incident cases. The consistency of results across analyses suggests that the observed associations are unlikely to be solely explained by reverse causation or by heterogeneity in sampling time. Nevertheless, we cannot fully exclude that EBNA1 levels partly reflect prior immune trajectories that are linked to both serology and disease progression. EBNA1 levels were measured at a single time point and against a single epitope (EBNA1 385–420), precluding assessment of temporal dynamics or responses to other EBV antigens. Although antibody responses to this epitope are strongly associated with MS susceptibility and overlap with a region homologous to GlialCAM/CRYAB (amino acids 386–406), they do not encompass other homology regions, such as that involving ANO2^7^. It remains uncertain whether antibody responses to this specific epitope correlate with T cell responses implicated in disability progression. Moreover, antibodies are not generally considered directly pathogenic in MS and may instead serve as biomarkers of underlying T cell-mediated immune processes. Antibody levels represent indirect markers of immune processes and do not allow differentiation between viral antigen burden, host immune competence or treatment-related effects on B-cell dynamics. Finally, residual confounding cannot be ruled out despite extensive covariate adjustment, particularly by unmeasured factors, such as comorbid conditions or infection history.

In conclusion, higher EBNA1 antibody levels were associated with a reduced risk of disability progression, with the strongest inverse association observed among individuals carrying both DRB1*15:01 and A*02:01. Our findings suggest that EBV-related immune markers do not operate as universal indicators of disease activity in MS. Rather, their prognostic relevance emerges within specific immunogenetic contexts, implying that host-virus interactions continue to shape disease course beyond disease onset.

## Supplementary material

10.1136/jnnp-2026-338964 online supplemental file 1

## Data Availability

Data are available upon reasonable request.
