# Supplementary material for: HLA-dependent association between EBNA1 antibody levels and disability progression in multiple sclerosis
Source: J Neurol Neurosurg Psychiatry. 2026 Jul 31;97(11):e338964. doi: 10.1136/jnnp-2026-338964 (PMC13691679; doi:10.1136/jnnp-2026-338964)
Supplement: Supplementary file 1 [file jnnp-97-11-s001.pdf]

Supplementary files

eTable 1. Sensitivity analysis limited to participants treated only with platform DMTs: Association between EBNA1 levels and risk of confirmed disability progression, overall and stratified by HLA-A*02:01 and HLA-DRB1*15:01 status.

eTable 2. Sensitivity analyses limited to DMT-treated participants: Association between EBNA1 levels and risk of confirmed disability progression, overall and stratified by HLA-A*02:01 and HLA-DRB1*15:01 status.

Table 3. Sensitivity analysis further adjusted for DMT at the time of blood sampling. Association between EBNA1 levels and risk of confirmed disability progression, overall and stratified by HLA-A*02:01 and HLA-DRB1*15:01 status.

eTable 4. Sensitivity analysis: incident cases with baseline as the time of blood sampling: Association between EBNA1 levels and risk of confirmed disability progression, overall and stratified by HLA-A*02:01 and HLA-DRB1*15:01 status.

eTable 5. Sensitivity analysis: incident cases with baseline as the time of blood sampling additionally adjusted for lifestyle factors: Association between EBNA1 levels and risk of confirmed disability progression, overall and stratified by HLA-A*02:01 and HLA-DRB1*15:01 status.

eTable 6. Disability status at the time of blood sampling across HLA strata.

eTable 7. Sensitivity analysis additionally adjusted for EDSS at blood sampling.

eFigure 1. Participant flow and eligibility for the analytic sample.

eFigure 2. Analytical framework for longitudinal EDSS analyses.

eFigure 3. Association between EBNA1 levels and risk of confirmed disability progression, stratified by HLA status.

eTable 1. Sensitivity analysis limited to participants treated only with platform DMTs: Association between EBNA1 levels and risk of confirmed disability progression, overall and stratified by HLA-A*02:01 and HLA-DRB1*15:01 status.

DRB1*15:01	A*02:01	N	Years (SD)	Outcome (%)	HR (95% CI)[1]	HR (95% CI)[2]
-/+	-/+	2523	6.7 (5.2)	1613 (63.9)	**0.91 (0.87-0.96)**	**0.93 (0.88-0.97)**
-	-	653	6.5 (5.1)	414 (63.4)	**0.87 (0.79-0.96)**	**0.88 (0.79-0.97)**
-	+	430	6.7 (5.0)	290 (67.4)	0.93 (0.84-1.03)	0.95 (0.85-1.05)
+	-	811	6.7 (5.3)	509 (62.8)	1.01 (0.92-1.10)	1.02 (0.93-1.12)
+	+	629	6.9 (5.3)	400 (63.6)	**0.85 (0.77-0.94)**	**0.86 (0.78-0.95)**

EBNA=Epstein-Barr virus nuclear antigen; HLA=human leukocyte antigen; HR=hazard ratio; CI=confidence interval; EDSS=expanded disability status scale; DMT=disease-modifying treatment. EBNA1 levels were standardized to z-scores; HRs represent the relative hazard per 1 standard deviation increase in EBNA1; [1]unadjusted; [2]adjusted for sex, age at baseline, ancestry, calendar year of diagnosis, disease phenotype, disease duration, and baseline EDSS. Bold values indicate statistical significance.

eTable 2. Sensitivity analyses limited to DMT-treated participants: Association between EBNA1 levels and risk of confirmed disability progression, overall and stratified by HLA-A*02:01 and HLA-DRB1*15:01 status.

DRB1*15:01	A*02:01	N	Years (SD)	Outcome (%)	HR (95% CI)[1]	HR (95% CI)[2]
-/+	-/+	4158	6.8 (5.2)	2647 (63.7)	**0.92 (0.89-0.96)**	**0.92 (0.89-0.96)**
-	-	1102	6.7 (5.1)	701 (63.6)	**0.92 (0.86-0.98)**	**0.91 (0.85-0.98)**
-	+	686	6.8 (4.9)	450 (65.6)	0.95 (0.87-1.03)	0.96 (0.88-1.22)
+	-	1326	6.8 (5.2)	847 (63.9)	0.96 (0.89-1.04)	0.97 (0.90-1.04)
+	+	1044	7.0 (5.3)	649 (62.2)	**0.86 (0.79-0.94)**	**0.87 (0.80-0.94)**

EBNA=Epstein-Barr virus nuclear antigen; HLA=human leukocyte antigen; HR=hazard ratio; CI=confidence interval; EDSS=expanded disability status scale; DMT=disease-modifying treatment. EBNA1 levels were standardized to z-scores; HRs represent the relative hazard per 1 standard deviation increase in EBNA1; [1]unadjusted; [2]adjusted for sex, age at baseline, ancestry, calendar year of diagnosis, disease phenotype, disease duration, baseline EDSS, and DMT exposure as a time-varying covariate. Bold values indicate statistical significance.

eTable 3. Sensitivity analysis further adjusted for DMT at the time of blood sampling. Association between EBNA1 levels and risk of confirmed disability progression, overall and stratified by HLA-A*02:01 and HLA-DRB1*15:01 status.

DRB1*15:01	A*02:01	N	Years (SD)	Outcome (%)	HR (95% CI)[1]	HR (95% CI)[2]
-/+	-/+	5706	6.7 (5.1)	3510 (61.5)	**0.93 (0.90-0.96)**	**0.93 (0.90-0.97)**
-	-	1472	6.6 (5.1)	897 (60.7)	**0.92 (0.86-0.98)**	**0.93 (0.87-0.99)**
-	+	951	6.9 (5.0)	589 (61.9)	0.96 (0.89-1.03)	0.96 (0.89-1.04)
+	-	1815	6.7 (5.2)	1121 (61.8)	0.97 (0.91-1.03)	0.97 (0.91-1.03)
+	+	1468	6.8 (5.2)	903 (61.5)	**0.86 (0.80-0.92)**	**0.88 (0.82-0.94)**

EBNA=Epstein-Barr virus nuclear antigen; HLA=human leukocyte antigen; HR=hazard ratio; CI=confidence interval; EDSS=expanded disability status scale; DMT=disease-modifying treatment. EBNA1 levels were standardized to z-scores; HRs represent the relative hazard per 1 standard deviation increase in EBNA1; [1]unadjusted; [2]adjusted for sex, age at baseline, ancestry, calendar year of diagnosis, disease phenotype, disease duration, baseline EDSS, and DMT exposure. Bold values indicate statistical significance.

eTable 4. Sensitivity analysis: incident cases with baseline as the time of blood sampling: Association between EBNA1 levels and risk of confirmed disability progression, overall and stratified by HLA-A*02:01 and HLA-DRB1*15:01 status.

DRB1*15:01	A*02:01	N	Years (SD)	Outcome (%)	HR (95% CI)[1]	HR (95% CI)[2]
-/+	-/+	1598	10.0 (5.1)	655 (41.0)	0.96 (0.89-1.04)	0.95 (0.88-1.03)
-	-	439	9.7 (5.1)	183 (41.7)	0.92 (0.80-1.06)	0.91 (0.79-1.05)
-	+	276	9.5 (4.9)	117 (42.4)	1.01 (0.87-1.17)	1.01 (0.86-1.18)
+	-	493	10.2 (5.1)	204 (41.4)	1.06 (0.91-1.24)	1.05 (0.91-1.23)
+	+	390	10.2 (5.3)	151 (38.7)	**0.86 (0.73-0.99)**	**0.84 (0.72-0.98)**

EBNA=Epstein-Barr virus nuclear antigen; HLA=human leukocyte antigen; HR=hazard ratio; CI=confidence interval; EDSS=expanded disability status scale; DMT=disease-modifying treatment. EBNA1 levels were standardized to z-scores; HRs represent the relative hazard per 1 standard deviation increase in EBNA1; [1]unadjusted; [2]adjusted for sex, age at baseline, ancestry, calendar year of diagnosis, disease phenotype, disease duration, baseline EDSS, and DMT exposure at baseline. Bold values indicate statistical significance.

eTable 5. Sensitivity analysis: incident cases with baseline as the time of blood sampling additionally adjusted for lifestyle factors: Association between EBNA1 levels and risk of confirmed disability progression, overall and stratified by HLA-A*02:01 and HLA-DRB1*15:01 status.

DRB1*15:01	A*02:01	N	Years (SD)	Outcome (%)	HR (95% CI)[1]	HR (95% CI)[2]
-/+	-/+	1598	10.0 (5.1)	655 (41.0)	0.96 (0.89-1.04)	0.95 (0.88-1.03)
-	-	439	9.7 (5.1)	183 (41.7)	0.92 (0.80-1.06)	0.90 (0.78-1.04)
-	+	276	9.5 (4.9)	117 (42.4)	1.01 (0.87-1.17)	1.00 (0.85-1.17)
+	-	493	10.2 (5.1)	204 (41.4)	1.06 (0.91-1.24)	1.05 (0.90-1.23)
+	+	390	10.2 (5.3)	151 (38.7)	**0.86 (0.73-0.99)**	**0.84 (0.71-0.98)**

EBNA=Epstein-Barr virus nuclear antigen; HLA=human leukocyte antigen; HR=hazard ratio; CI=confidence interval; EDSS=expanded disability status scale; DMT=disease-modifying treatment. EBNA1 levels were standardized to z-scores; HRs represent the relative hazard per 1 standard deviation increase in EBNA1; [1]unadjusted; [2]adjusted for sex, age at baseline, ancestry, calendar year of diagnosis, disease phenotype, disease duration, baseline EDSS, DMT exposure at baseline, past infectious mononucleosis, other autoimmune disease, educational attainment, smoking status, alcohol use, sun exposure, body mass index, and physical activity. Bold values indicate statistical significance.

eTable 6. Disability status at the time of blood sampling across HLA strata

DRB1*15:01	A*02:01	N	Median EDSS (IQR)
-	-	1472	3.0 (1.5-6.0)
-	+	951	3.0 (1.5-6.0)
+	-	1815	3.0 (1.5-6.0)
+	+	1468	3.0 (1.5-6.0)

HLA=human leukocyte antigen; EDSS=expanded disability status scale; IQR=interquartile range. Overall median EDSS at blood sampling was 3.0 (IQR 1.5-6.0).

eTable 7. Sensitivity analysis additionally adjusted for EDSS at blood sampling.

DRB1*15:01	A*02:01	N	Years (SD)	Outcome (%)	HR (95% CI)[1]	HR (95% CI)[2]
-/+	-/+	5706	6.7 (5.1)	3510 (61.5)	**0.93 (0.90-0.96)**	**0.96 (0.92-0.99)**
-	-	1472	6.6 (5.1)	897 (60.7)	**0.92 (0.86-0.98)**	0.98 (0.91-1.05)
-	+	951	6.9 (5.0)	589 (61.9)	0.96 (0.89-1.03)	0.96 (0.88-1.04)
+	-	1815	6.7 (5.2)	1121 (61.8)	0.97 (0.91-1.03)	0.98 (0.91-1.05)
+	+	1468	6.8 (5.2)	903 (61.5)	**0.86 (0.80-0.92)**	**0.90 (0.84-0.97)**

EBNA=Epstein-Barr virus nuclear antigen; HLA=human leukocyte antigen; HR=hazard ratio; CI=confidence interval; EDSS=expanded disability status scale; DMT=disease-modifying treatment. EBNA1 levels were standardized to z-scores; HRs represent the relative hazard per 1 standard deviation increase in EBNA1; [1]unadjusted; [2]adjusted for sex, age at baseline, ancestry, calendar year of diagnosis, disease phenotype, disease duration, baseline EDSS, and EDSS at blood sampling. Bold values indicate statistical significance.

eFigure 1. Participant flow and eligibility for the analytic sample.

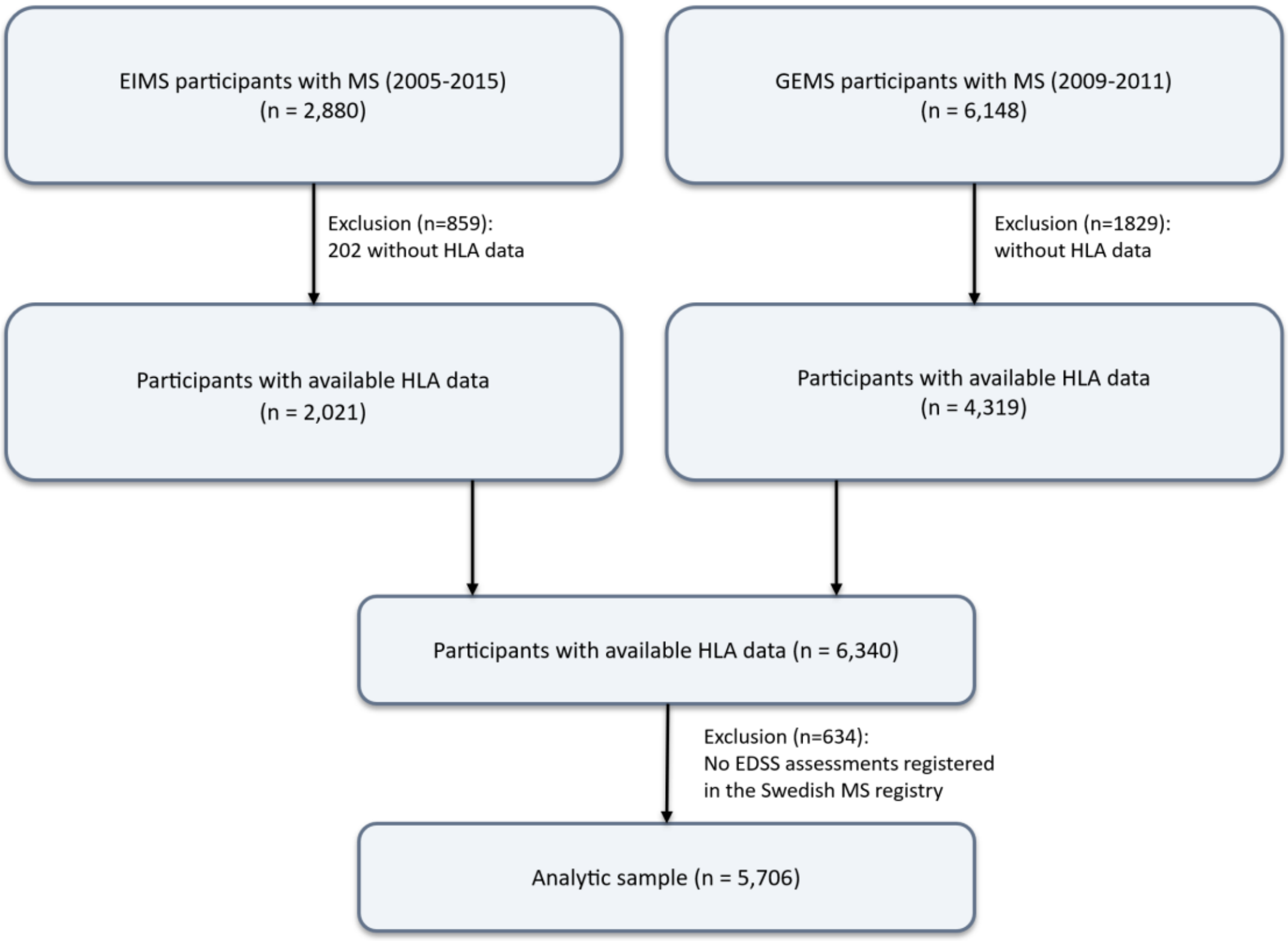

eFigure 2. Analytical framework for longitudinal EDSS analyses

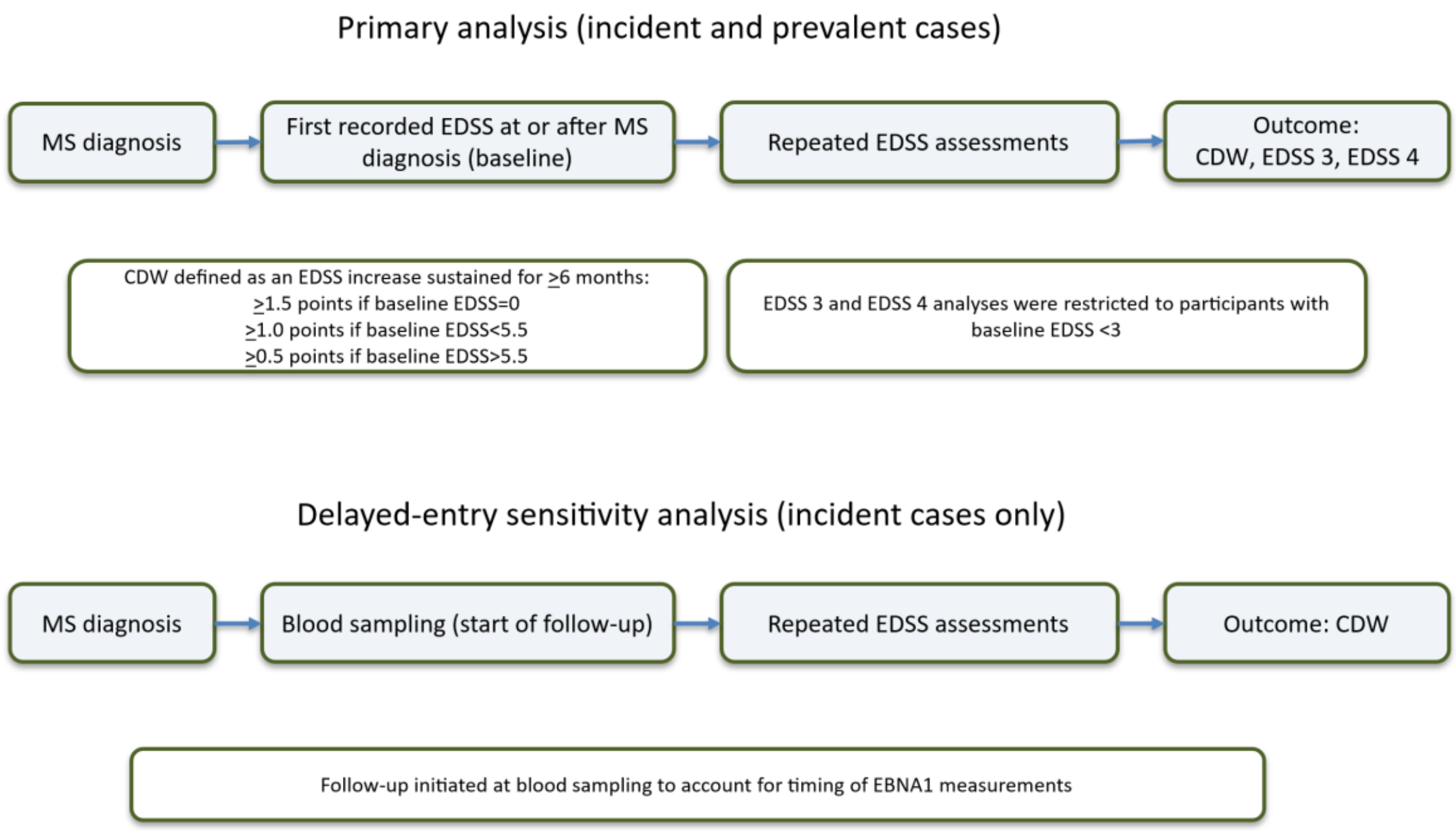

eFigure 3. Association between EBNA1 levels and risk of confirmed disability progression, stratified by HLA status.

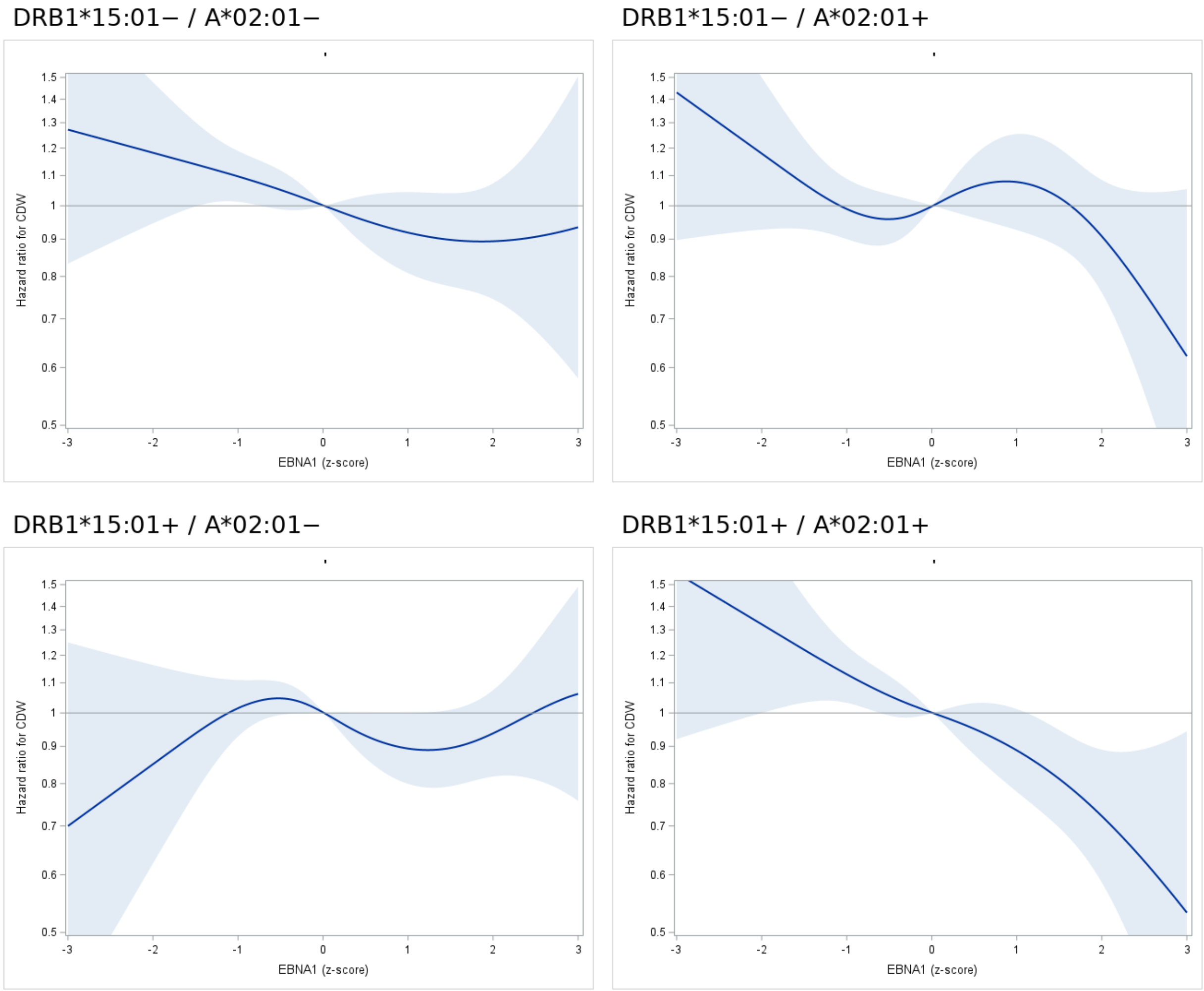

EBNA=Epstein-Barr virus nuclear antigen; EDSS=expanded disability status scale. Hazards ratios for confirmed disability progression across standardized EBNA1 levels are shown relative to the median EBNA1 value in the total cohort (HR=1). EBNA1 levels were standardized to z-scores. Solid line represents adjusted hazard ratios estimated from Cox proportional hazards models; shaded area indicates 95% confidence intervals. Models were adjusted for sex, age at baseline, ancestry, calendar year of diagnosis, disease phenotype, disease duration, and baseline EDSS.
